# Arginine within a specific motif near the N‐terminal of FimY is critical for the maximal production of type 1 fimbriae in *Salmonella enterica* serovar Typhimurium

**DOI:** 10.1002/mbo3.846

**Published:** 2019-04-16

**Authors:** Nan‐Ling Kuan, Kuang‐Sheng Yeh

**Affiliations:** ^1^ Biology Division Animal Health Research Institute New Taipei City Taiwan; ^2^ Department of Veterinary Medicine, School of Veterinary Medicine, College of Bioresources and Agriculture National Taiwan University Taipei Taiwan; ^3^ National Taiwan University Veterinary Hospital Taipei Taiwan

**Keywords:** *fimY*, PilZ domain, *Salmonella* Typhimurium, type 1 fimbriae

## Abstract

An important *Salmonella* serovar for both human and animals *Salmonella* Typhimurium possesses 13 gene clusters that have the potential to produce fimbrial structure, among which the type 1 fimbriae with the binding specificity to mannose residue is the most commonly found type. Six structural genes and five regulatory genes comprise the *fim* gene cluster that is responsible for the production of type 1 fimbriae in *S*. Typhimurium. The *fimY* gene encodes a positive regulator for type 1 fimbrial expression since a deletion in *fimY* abolished the production of fimbriae. The N‐terminal portion of FimY contains amino acid residues that exhibit some similarity as those found in the proteins possessing the PilZ domain, which is engaged in cyclic di‐GMP binding. A *fimY* allele that had a change from arginine to alanine at position 7 (R7A) or 7 and 11 (R7/11A) generated by site‐directed mutagenesis in a ^6^RRERH^11^R motif near N‐terminal, when cloned in pACYC184 and transformed into a *fimY*‐deleted strain, decreased the expression of *fimA* and *fimZ*. The number of type 1 fimbriae in these two transformants was also less than those of the other transformants that contained different *fimY* alleles in pACYC184 when observed in electron microscopy. However, changing from arginine to alanine at position 11 (R11A) remained the same as the wild‐type *fimY* allele. It is likely that the arginine at the 7th position of FimY is critical for its maximal activating activity upon *fimZ*. Another motif ^83^DI^85^SLWIEK^91^G motif did not affect the function of FimY. Although FimY has the two aforementioned motifs, which contain some amino acids that are present within those of the PilZ domain proteins, secondary structure prediction analysis did not reveal that FimY has a conformation commonly observed in PilZ‐like proteins. Therefore, FimY and PilZ domain proteins are not homologs. Further investigation for a detailed analysis of FimY is thus warranted.

## 1 INTRODUCTION

**Figure 1:**
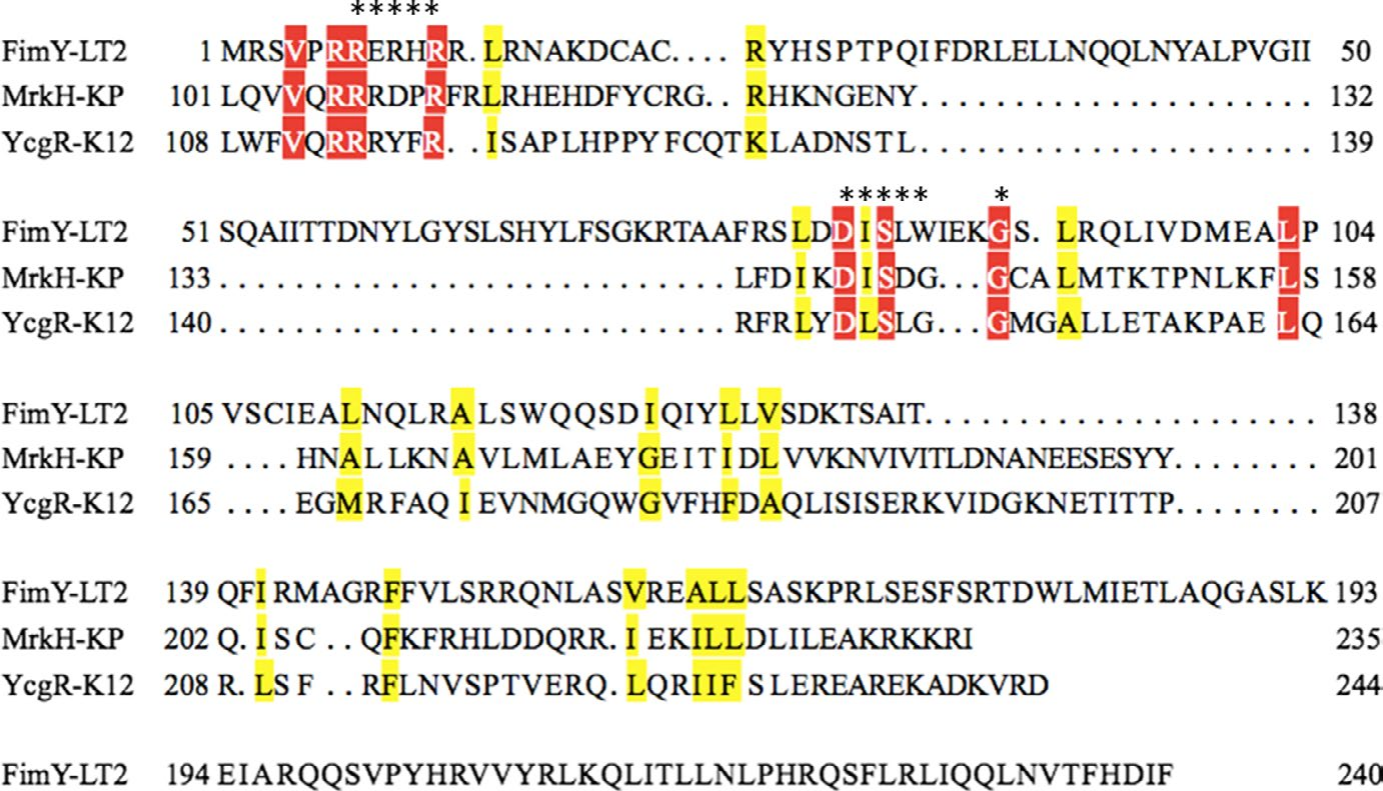
Conservation of PilZ‐containing domain proteins. Residues that show strict identity are written in white characters and highlighted in red. Similarity is indicated with black characters and highlighted in yellow. Residues that are crucial for c‐di‐GMP binding in the PilZ domain are marked with an asterisk. Protein names and microorganisms are as follows: FimY‐LT2, FimY of *Salmonella* Typhimurium LT2; MrkH‐KP, MrkH of *Klebsiella pneumoniae* AJ218; YcgR‐K12, YcgR of *Escherichia coli* K‐12

*Salmonella* belongs to the member of the family *Enterobacteriaceae* and is an important zoonotic agent of public health concern. Nontyphoid *Salmonella* accounted for over 59,000 deaths and they were with the highest rank for disability adjusted life years among the foodborne disease hazards in 2010 (Kirk et al., 2015). *Salmonella enterica* contains more than 2,579 serovars while *S*. Typhimurium is one of the major serovars of *Salmonella* that account for infections in human and animals (CDC, 2011). Adhesion to the host cell is the first step to establish infection for many bacteria including *S.* Typhimurium. Adhesion molecules on the surface of bacteria or conjugated on the shaft of a structure called fimbria have been implicated in engaging such an adherent event. In addition, signal transduction in bacteria can also be mediated by adhesion to the host cell (Moorthy, Keklak, & Klein, 2016). Fimbriae are hair‐like appendages present on the outer membrane of many bacteria. *S.* Typhimurium has been documented to possess 13 different fimbrial gene clusters that may have the potential to produce fimbriae (McClelland et al., 2001). Among these fimbriae types, type 1 fimbriae with the binding specificity to mannosylated residue is frequently found and is also referred to as the common fimbriae (Duguid, Anderson, & Campbell, 1966). The *fim* gene cluster in the genome of *S.* Typhimurium is responsible for the phenotypic expression of type 1 fimbriae. The *fim* gene cluster consists of genes for structure, biosynthesis, and those for regulation. FimA, FimI, FimF, and FimH are fimbrial structural subunits that incorporate each other to form a fimbrial shaft. Fimbrial subunits are assembled and anchored in the outer membrane of bacteria by chaperone protein FimC and usher protein FimD, respectively (Clegg & Swenson, 1994). The genes *fimZ*, *fimY*, *fimW*, *stm0551,* and *fimU* have been shown to regulate type 1 fimbrial production through a complicated circuit involving both the transcriptional and translational levels and protein–protein interaction as well (Tinker & Clegg, 2001; Tinker, Hancox, & Clegg, 2001; Yeh, Hancox, & Clegg, 1995). FimY is a positive regulator for type 1 fimbrial expression in *S*. Typhimurium. The C‐terminal part of FimY possesses a helix‐turn‐helix motif and FimY was shown to bind to the promoter region of *fimZ* (Wang, Hsu, Huang, Lin, & Yeh, 2014); however, some aspects of its mechanism in terms of fimbrial regulation remains to be elucidated. For example, the members of the PilZ family regulator, such as YcgR of *Escherichia coli* and MrkH of *Klebsiella pneumoniae* (Ryjenkov, Simm, Romling, & Gomelsky, 2006; Wilksch et al., 2011), often contain two conserved domains RRxxxR and DxSxxG (x designates any amino acid), which involve in binding cyclic di‐GMP (c‐di‐GMP), a second messenger that controls many physiological processes of bacteria (Jenal, Reinders, & Lori, 2017). According to the protein sequence alignment, the N‐terminal of FimY also possesses some amino acids that are present in these motifs (Figure 1). FimY harbors a ^6^RRERH^11^R and a ^83^DI^85^SLWIEK^91^G motif, with the second motif having extra three amino acids as compared with the DxSxxG motif; yet, its role as a c‐di‐GMP–binding protein has not been reported. Interestingly, a gene, *stm0551*, located just upstream of *fimY* was proven to encode a phosphodiesterase with an EAL domain whose function is to degrade c‐di‐GMP (Wang, Hsu, Huang, & Yeh, 2012). Nevertheless, there is no protein resembling a diguanylate cyclase to synthesize c‐di‐GMP within the *fim* gene cluster.

The present study reported here attempts to uncover the potential roles of ^6^RRERH^11^R and ^83^DI^85^SLWIEK^91^G of FimY and investigate how it may affect type 1 fimbrial expression in *S*. Typhimurium. We found that the 7th arginine at the ^6^RRERH^11^R motif of FimY may affect the production of type 1 fimbriae by activating *fimZ* and subsequently resulting in the fimbrial major subunit gene *fimA* expression.

## 2 MATERIALS AND METHODS

### 2.1 Bacterial strains, plasmids, and culture conditions

Table 1 lists the bacterial strains and plasmids used in this study. All strains were grown in Luria–Bertani (LB) broth or on an LB agar at 37°C. For transformant selection, media may be required to be supplemented with antibiotics such as ampicillin (100 μg/ml), kanamycin (25 μg/ml), or chloramphenicol (25μg/ml). *Escherichia coli* strains were carried out for plasmid construction, plasmid purification, and cloning using standard techniques. For the type 1 fimbriae inducing condition, all *S*. Typhimurium strains were cultured in static broth at 37°C for 48 hr.

**Table 1:** Bacterial strains and plasmids used in this study

Strain or plasmid	Description	Abbreviation	Source or reference
Strains			
*Salmonella* Typhimurium			
LB5010	a *S.* Typhimurium LT2 derivative, wild type, fimbriate with the complete *fim* gene cluster	Wild type	
LB5010Δ*fimY*	a *fimY*‐deleted strain	Δ*fimY*	This study
LB5010Δ*fimY* (*fimY*)	a *fimY*‐deleted strain transformed with p*fimY*	Δ*fimY* (*fimY*)	This study
LB5010Δ*fimY* (pACYC184)	a *fimY*‐deleted strain transformed with pACYC184	Δ*fimY* (pACYC184)	This study
LB5010Δ*fimY* (*fimY*_R7A_)	a *fimY*‐deleted strain transformed with p*fimY*_R7A_	R7A	This study
LB5010Δ*fimY* (*fimY*_R11A_)	a *fimY*‐deleted strain transformed with p*fimY*_R11A_	R11A	This study
LB5010Δ*fimY* (*fimY*_R7/11A_)	a *fimY*‐deleted strain transformed with p*fimY*_R7/11A_	R7/11A	This study
LB5010Δ*fimY* (*fimY*_DSG_)	a *fimY*‐deleted strain transformed with p*fimY*_DSG_	DSG	This study
LB5010Δ*fimY* (*fimY*_MUT_)	a *fimY*‐deleted strain transformed with p*fimY*_MUT_	MUT	This study
*Escherichia coli*			
One Shot® TOP10	a chemically competent cell strain for molecular cloning		Invitrogen
Plasmids			
pKD13	A template plasmid for gene inactivation, Kan^r^		Datsenko & Wanner, 2000
pKD46	The plasmid expressing λ Red recombinase; Amp^r^		Datsenko & Wanner, 2000
pACYC184	Cloning vector; Tet^r^; and Cm^r^		ATCC
pSTBLUE‐1	Cloning vector; Kan^r^; and Amp^r^		Novagen
p*fimY*	A complete *fimY* coding sequence cloned into pACYC184; Cm^r^		This study
p*fimY*_R7A_	A *fimY* coding sequence with R7A cloned into pACYC184; Cm^r^		This study
p*fimY*_R11A_	A *fimY* coding sequence with R11A cloned into pACYC184; Cm^r^		This study
p*fimY*_R7/11A_	A *fimY* coding sequence with R7/11A cloned into pACYC184; Cm^r^		This study
p*fimY*_DSG_	A *fimY* mutant sequence with D83A, S85A, and G91A cloned into pACYC184; Cm^r^		This study
p*fimY*_MUT_	A *fimY* mutant sequence with R7/11A, D83A, S85A, and G91A cloned into pACYC184; Cm^r^		This study

### 2.2 Construction of a *S*. Typhimurium  *fimY‐*deleted strain

The *S*. Typhimurium  *fimY*‐deleted strain was constructed by a one‐step gene inactivation method (Datsenko & Wanner, 2000). Kanamycin resistance gene from pKD13 tagged with a flanking sequence of the *fimY* gene was generated by a polymerase chain reaction (PCR) technique using *fimY*‐P1 and *fimY*‐P4 and transformed by electroporation into *S*. Typhimurium LB5010 that has previously been transformed with pKD46, the plasmid that encodes a λ red recombinase. The primers and PCR conditions are listed in Table 2. All transformants were grown on LB agar supplemented with kanamycin. The constructed mutants were verified by PCR with primers located in the flanking sequence of the *fimY* gene.

**Table 2:**
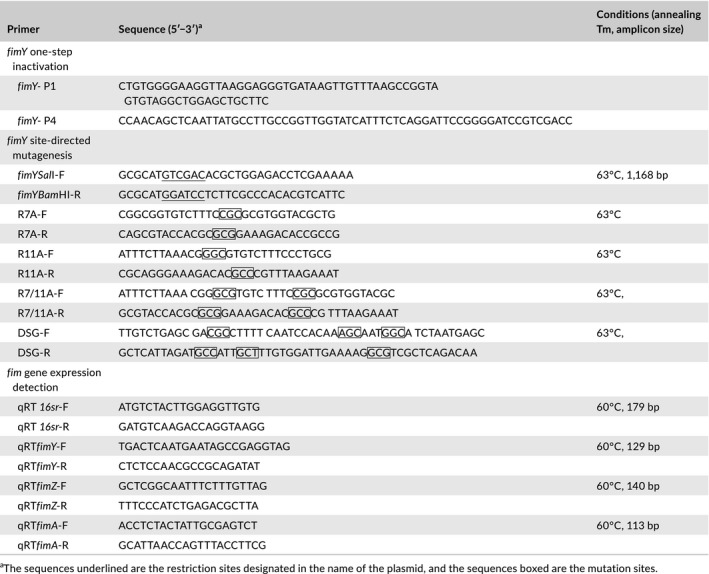
Primers used in this study

### 2.3 Construction of the recombinant plasmids possessing the *fimY* PilZ‐like domain mutant alleles

The mutant alleles of *fimY* were generated by site‐directed mutagenesis using an overlapping extension PCR with LB5010 strain genomic DNA (Ho, Hunt, Horton, Pullen, & Pease, 1989). The primers are listed in Table 2. Briefly, *fimYSal*I‐F and R7A‐R were used to amplify the first DNA fragment, while the second DNA fragment was amplified using *fimYBam*HI‐R and R7A‐F primers. Ligation of these two DNA fragments with two overlapping ends was achieved with *fimYSal*I‐F and *fimYBam*HI‐R primers. The PCR conditions consisted of initial denaturation at 95°C for 30 s, followed by 35 cycles at 95°C for 30 s, 63°C for 30 s, and 72°C for 45 s using KOD DNA polymerase (Novagen, Madison, WI). The ligated fragments were cloned into the pSTBLUE‐1 blunt vector (Novagen) and sequenced to determine whether the codon encoding the specific amino acid had been replaced with alanine. The other *fimY* alleles were constructed with the same methods, except that all five amino acids of *fim*PilZ‐like domain mutant were accomplished by primers DSG‐F and DSG‐R using p*fimY*_R7/11A_ as the template. Table 3 lists the *fimY* mutant alleles by site‐directed mutagenesis. For the complementation test, the ligated fragment was cloned into the pACYC184 vector and transformed into LB5010Δ*fimY* strain by electroporation, and the transformants were tested for the ability to produce type 1 fimbriae as described below. Confirmation that only appropriate sequences had been mutated was performed by nucleotide sequence analysis (Mission Biotech, Taipei, Taiwan).

**Table 3:** Site‐directed mutagenesis of *fimY* PilZ‐like domain

Plasmid	FimY amino acid sequence
p*fimY*	^7^RERH^11^R ^83^DI^85^SLWIEK^91^G
p*fimY*_R7A_	^7^AERH^11^R ^83^DI^85^SLWIEK^91^G
p*fimY*_R11A_	^7^RERH^11^A ^83^DI^85^SLWIEK^91^G
p*fimY*_R7/11A_	^7^AERH^11^A ^83^DI^85^SLWIEK^91^G
p*fimY*_DSG_	^7^RERH^11^R ^83^AI^85^ALWIEK^91^A
p*fimY*_MUT_	^7^AERH^11^A ^83^AI^85^ALWIEK^91^A

### 2.4 Electron microscopy

*Salmonella* strains were prepared for electron microscopy using the negative staining procedure. Briefly, the bacterial culture was resuspended in ddH_2_O and mixed with an equal volume of sodium phosphotungstate (2%). A drop of the suspension was placed on a glow‐discharged carbon‐coated copper grid, and surplus fluid was removed with filter papers. The grids were air‐dried and observed with a JEOL JEM‐1400 transmission electron microscope (JEOL Ltd, Tokyo, Japan) operated at 60 or 80 kV.

### 2.5 Detection of type 1 fimbriae by microplate yeast agglutination test

To analyze the expression ability of type 1 fimbriae of *Salmonella* strains, a microplate agglutination test (MAT) using yeast cells was performed in this study. All the *Salmonella* strains were cultured in 10 ml LB broth at 37°C for 48 hr statically, followed by centrifugation at 3,000 rpm for 10 min. MAT was performed as follows: First, the bacterial suspensions were adjusted to OD_600_ 1 ± 0.05, followed by twofold serial dilution in phosphate‐buffered saline (PBS), and 100 µl of each dilution preparation was added in the well of a 96‐well round‐bottom microplate. Then, an equal volume (100 µl) of 0.5% *Saccharomyces cerevisiae* (Sigma‐Aldrich, Darmstadt, Germany) was added to each well and mixed. The plates were incubated at 37°C for 30 min. The highest dilution of the suspensions showing agglutination was defined as the titer end point.

### 2.6 Quantitative RT‐PCR analysis

Total RNA was extracted and purified from bacterial strains cultured in static broth with the ZR Fungal/Bacterial RNA Miniprep™ kit (Zymo Research, Irvine, CA) according to the manufacturer's instructions. To remove the residual DNA, all the extractions were treated with RNase‐free DNase I (ThemoFisher Scientific, Waltham, MS). The purified RNA (0.1 μg) was converted to cDNA using iScript™ cDNA Synthesis kit (Bio‐Rad Laboratories, Hercules, CA), and transcribed into mRNA using iTaq™ universal SYBR^®^ Green supermix (Bio‐Rad Laboratories). Transcription of *fimA*, *fimY*, *fimZ*, and 16s rRNA as an internal control were detected by quantitative RT‐PCR. Cycling conditions were performed using CFX Connect™ Real‐Time System (Bio‐Rad Laboratories) as follows: 95°C for 3 min followed by 49 cycles of 95°C for 10 s and 60°C for 30 s. Melting curves and nontemplate controls were included to detect any primer dimerization or other artifacts. The mRNA transcript levels were analyzed by the software Bio‐Rad CFX Manager 3.1 (Bio‐Rad Laboratories).

### 2.7 Secondary structure analysis

A simple secondary structure prediction program, Profile network from Heidelberg (PHD) in NPS@: Network Protein Sequence Analysis [available at https://npsa-prabi.ibcp.fr/cgi-bin/npsa_automat.pl?page=/NPSA/npsa_phd.html] (Combet, Blanchet, Geourjon, & Deleage, 2000; Rost & Sander, 1993) was used to predict the secondary structures of FimY, MrkH, DgrA, and VCA0042. To predict the distributions of the secondary structures (e.g., α‐helix, random coil, or β‐sheet), 100 amino acids starting from ^6^RRERH^11^R of FimY, ^106^RRRDP^111^R of MrkH, ^11^RRAHP^16^R of DgrA, and ^134^QLRKEP^140^R of VCA0042 were analyzed using PHD.

## 3 RESULTS

### 3.1 The electron microscopy

**Figure 2:**
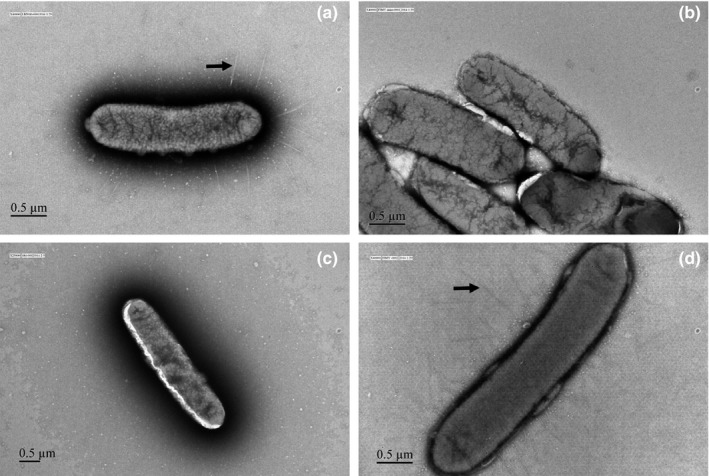
Observation of type 1 fimbriae by electron microscopy. (a) *S*. Typhimurium LB5010 wild‐type strain produced type 1 fimbriae on the outer membrane of the cell. (b) Δ*fimY* did not produce type 1 fimbriae. (c) There are no type 1 fimbriae present in the Δ*fimY* (pACYC184). (d) Δ*fimY* (*fimY*) resumed the ability to express type 1 fimbriae. Bacterial cells were negatively stained with 2% of phosphotungstic acid (60,000 x–80,000 x). Fimbriae are indicated by arrow

**Figure 3:**
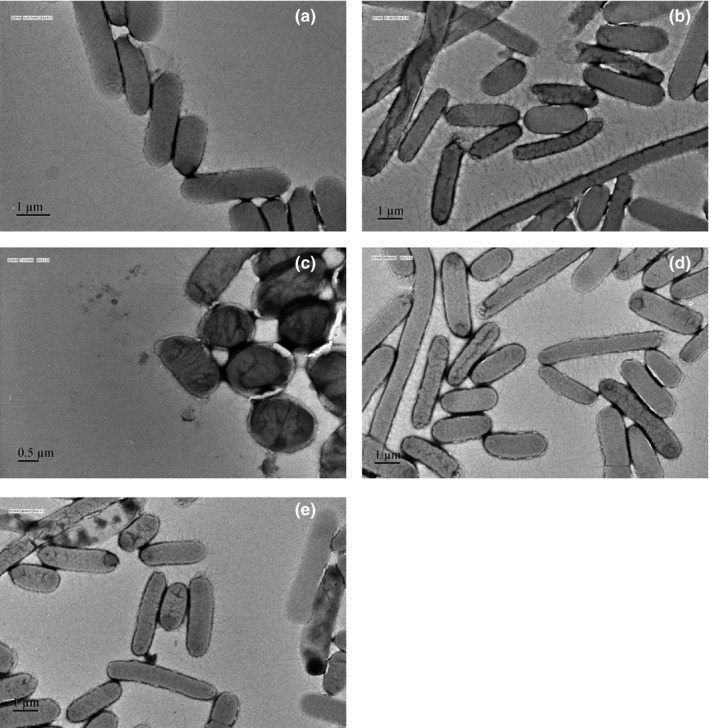
Observation of type 1 fimbrial expression in the Δ *fimY* strains that harbored plasmids expressing different *fimY* allels by electron microscopy. (a) R7A strain (b). R11A strain (c). R7/11A strain (d). DSG strain (e). MUT strain. All the strains produced type 1 fimbriae, whereas R7A and R7/11A strains produced comparatively less fimbriae than the others. Bacterial cells were negatively stained with 2% of phosphotungstic acid (60,000 x‐80,000 x)

All *Salmonella* strains were observed for the phenotypic expression of type 1 fimbriae by electron microscopy. *S*. Typhimurium LB5010 wild type and the Δ*fimY* (*fimY*) strains produced type 1 fimbrial appendages, whereas no fimbrial structures were detected in the Δ*fimY* strain and its transformant containing pACYC184 cloning vector (Figure 2). The Δ*fimY* strain transformants which received recombinant plasmids possessing different *fimY* alleles demonstrated different levels of type 1 fimbriae. It was shown that the R7A, R7/11A, and MUT strains revealed relatively fewer numbers of type 1 fimbriae than those that possessed other *fimY* alleles (Figure 3).

### 3.2 Detection of type 1 fimbriae by microplate yeast agglutination test

**Figure 4:**
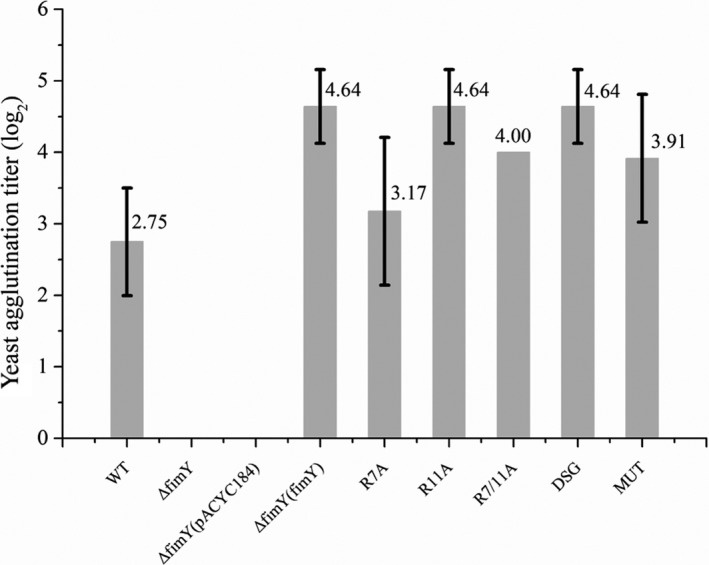
Detection of type 1 fimbriae by microplate yeast agglutination test. The numbers indicate the geometric average of the titer end point (*N* = 3) of different *Salmonella* strains

Microplate yeast agglutination test was used to quantify the type 1 fimbriae expression in all *Salmonella* strains. Figure 4 demonstrated the base two logarithms of a geometric average titer for each sample. The Δ*fimY* and Δ*fimY* (pACYC184) strains did not produce type 1 fimbriae and no titer was detected. The titer of the Δ*fimY* (*fimY*) was 4.64, which was higher than that of the wild type (2.76). The titer of other Δ*fimY* transformants were as follows: R11A and DSG were the same as Δ*fimY* (*fimY*), R7/11A and MUT were nearly 4, and the lowest was R7A (3.17). Compared with the Δ*fimY* (*fimY*), whose titer was considered as a baseline, the decrease in the titer of R7A was over 50%, while R7/11A and MUT were close to 36%.

### 3.3 Quantitative RT‐PCR analysis

**Figure 5:**
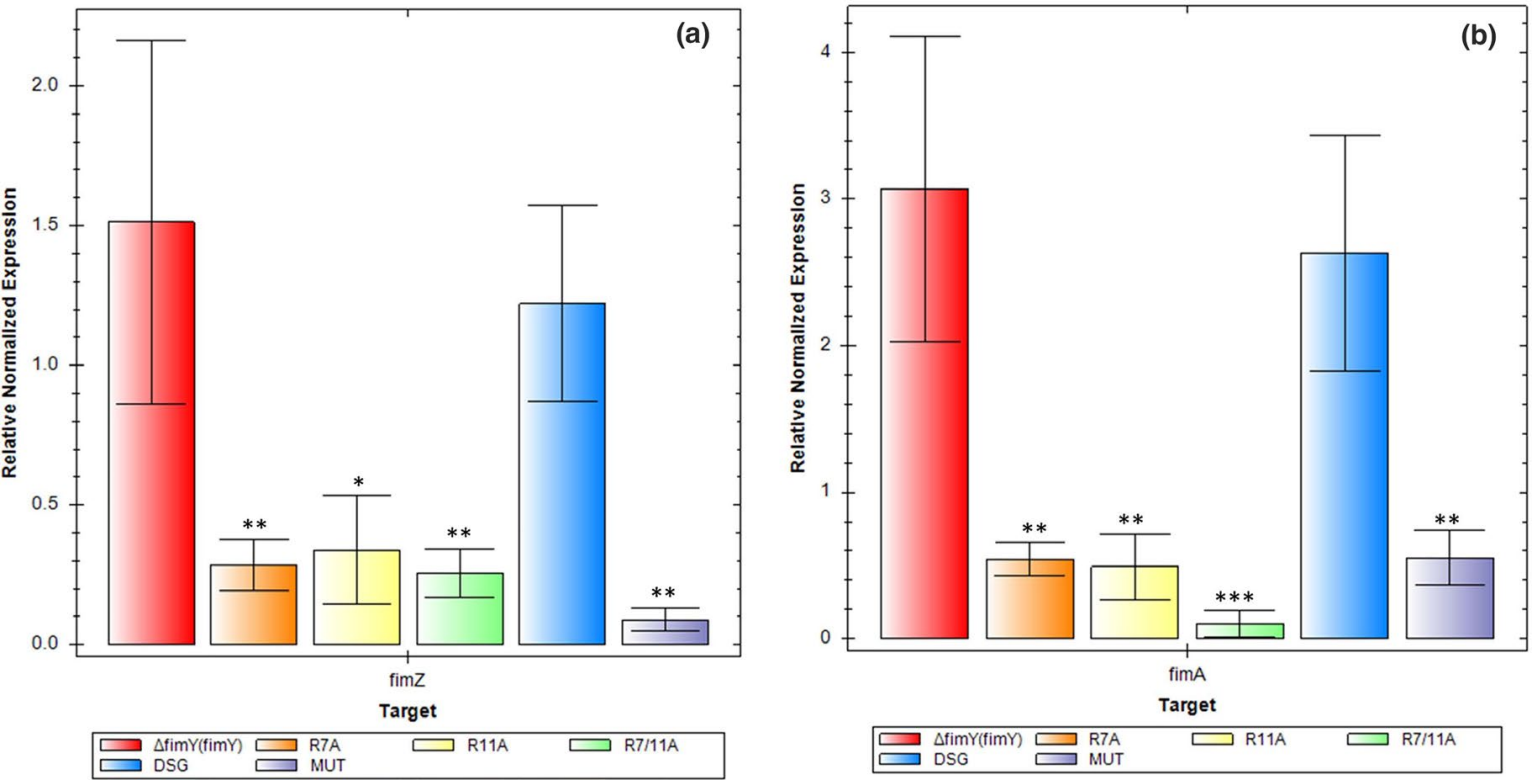
Quantitative real‐time polymerase chain reaction analysis of *fimA* and *fimZ* of different *S*. Typhimurium strains. The expression of (a) *fimZ* and (b) *fimA* was calculated using the ^ΔΔ^Ct method and designed as the fold change compared with the geometric mean expression level of the Δ*fimY* (*fimY*) strain in triplicate. A *p* value < 0.05 was considered to represent a significant difference. *p < 0.05; **p < 0.01; ***p < 0.001

The DSG strain exhibited about 0.8‐fold of the *fimZ* expression as compared to the control strain Δ*fimY* (*fimY*), while the other strains R7A, R11A, R7/11A, and MUT produced less *fimZ* expression. Similar profile was observed on *fimA* expression, the DSG strain exhibited relatively similar *fimA* expression as the Δ*fimY* (*fimY*) strain and the others exhibited the *fimA* level significantly lower than that of the Δ*fimY* (*fimY*) strain (Figure 5).

### 3.4 Secondary structure analysis

**Figure 6:**
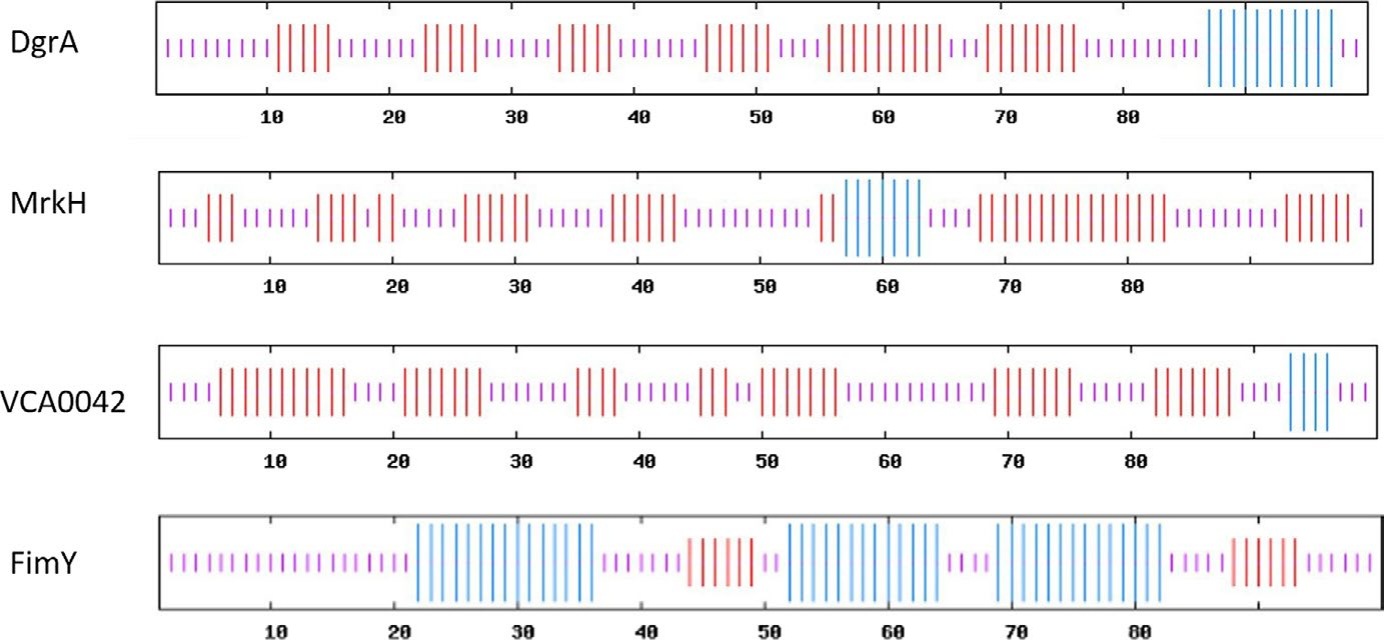
Secondary structure prediction. Protein size of 100 amino acids beginning from ^6^RRERH^11^R of FimY in *Salmonella* Typhimurium (accession No.: AAL19504), ^106^RRRDP^111^R of MrkH in *Klebsiella pneumoniae* (accession No.: AEO27488), ^11^RRAHP^16^R of DgrA in *Caulobacter crescentus* (accession No.: AE005673.1), and ^134^QLRKEP^140^R of VCA0042 in *Vibrio cholerae* (accession No.: NP_232443) were analyzed using PHD to predict their secondary structures; red: β‐strand; pink: random coli; blue: α‐helix.

If FimY serves as a PilZ domain‐like protein in *S*. Typhimurium, the secondary structure prediction program would reveal similarities between the configurations of FimY and other well‐known PilZ domain proteins, such as MrkH of *K*. *pneumoniae*, DgrA of *Caulobacter crescentus*, and VCA0042 of *Vibrio cholerae* (Heidelberg et al., 2000; Ryjenkov et al., 2006; Wilksch et al., 2011). Figure 6 shows that the secondary structure pattern of FimY did not match that of the other well‐documented PilZ domain proteins—the RRxxxR at the N‐terminal followed by 6–7 β‐strands forming a β‐barrel and a C‐terminal α‐helix (Amikam & Galperin, 2006). However, the N‐terminal of FimY only contained an α‐helix.

## 4 DISCUSSION

*S*. Typhimurium possesses three well‐defined processes which are regulated by c‐di‐GMP, they are flagellar‐based motility, curli fimbriae formation, and cellulose production (Kader, Simm, Gerstel, Morr, & Romling, 2006; Ryjenkov et al., 2006; Solano et al., 2002; Zogaj, Nimtz, Rohde, Bokranz, & Romling, 2001). Previous studies have demonstrated that c‐di‐GMP binding depends on residues in the RRxxxR and DxSxxG sequence motif conserved in the PilZ domains (Christen et al., 2007; Merighi, Lee, Hyodo, Hayakawa, & Lory, 2007). Like most of the *Enterobacteriaceae* members, *S*. *typhimurium* has two PilZ domain proteins, YcgR and BcsA, which control motility and cellulose production, respectively (Pultz et al., 2012). Within the elements of the *fim* gene cluster of *S*. Typhimurium only *stm0551* had been described to involve in c‐di‐GMP metabolism, whose product is a phosphodiesterase to degrade c‐di‐GMP (Wang et al., 2012). No PilZ domain protein had been reported previously. In this study, we found that the amino acid of FimY does possess a RRxxxR motif and another DxSxxxxxG sequence motif; however, the function of these motifs has not been explored. Therefore, it is interesting to investigate the function of these motifs in terms of type 1 fimbrial production.

Since FimY is a positive regulator for type 1 fimbrial production, a *fimY*‐deleted strain Δ*fimY* did not possess fimbrial appendages. Transforming an intact *fimY* allele cloned in pACYC184 into the Δ*fimY* restored the fimbriate positive phenotype. However, transformation of the plasmids possessing the *fimY* alleles with amino acid changes in different positions enabled Δ*fimY* to produce different levels of fimbrial structures. Comparatively, the amount of fimbriae found in R7A, R7/11A, and MUT strains was less than those of the other transformants. Since the R11A strain did not decrease the amount of fimbriae, it is highly possible that the change from arginine to alanine at position 7, but not 11, in FimY had an impact on type 1 fimbrial expression. In another study, DgrA of *Caulobacter crescentus* controls flagellar motor function and it functions as a c‐di‐GMP–binding protein; mutation of R11A/R12A in the ^11^RRxxxR^16^ motif of DgrA abolished the binding ability of c‐di‐GMP (Christen et al., 2007). It was postulated that the side chains of arginine participate in hydrogen bond or in electrostatic interactions with c‐di‐GMP, as the similar to that in the allosteric binding site of the diguanylate cyclases such as PleD and DgcA (Chan et al., 2004; Christen et al., 2006). In addition, the positive charged head groups of arginine are required for transient binding to the phosphate groups of c‐di‐GMP (Shoemaker, Portman, & Wolynes, 2000). The R7A and R7/11A strains decreased the production of fimbriae and R7A exhibited a decreased microplate yeast agglutination titer; these findings did somehow underscore the important role of R7 in FimY. As to the yeast agglutination test, FimH protein incorporated on the fimbrial shaft mediates the agglutination of yeast cell. The titer end point of the microplate agglutination test should correlate with the number of type 1 fimbriae on *S*. *typhimurium*. The wild‐type strain showed an agglutination titer of 2.75 while Δ*fimY* and Δ*fimY* (pACYC184) strains were both not fimbriate, hence no agglutination was detected. The reason that Δ*fimY* (*fimY*) strain exhibited a higher agglutination titer than that of the wild‐type strain (4.64 vs. 2.75) may be due to the copy number effect of the pACYC184. In the regulatory network of type 1 fimbriae in *S*. *typhimurium*, *fimY* may act upstream of *fimZ*; that is to say, *fimY* activates *fimZ*, and in turn *fimZ* activates *fimA*, resulting in the production of fimbriae (Wang et al., 2014; Zeiner, 2012). Many *fimY* transcripts in Δ*fimY* (*fimY*) strain should be responsible for the mass production of *fimA* through the *fimY→fimZ→fimA* pathway, leading to a high agglutination titer.

The DSG strain contains an intact ^6^RRERHR^11^ motif but with aspartic acid, serine, and glycine at 83, 85, and 91 position, respectively, substituted with alanine at the ^83^DI^85^SLWIEK^91^G motif. A previous study indicated that the aspartic acid and glycine residues of DxSxxG motif are required for c‐di‐GMP binding (Christen et al., 2007). The R11A and DSG strains both had the same agglutination titer as the ΔfimY (fimY) strain and these two strains also produced similar number of fimbriae as the ΔfimY (fimY) strain. Besides, the *fimZ* and *fimA* expression of DSG strain were close to that of the Δ*fimY* (*fimY*) strain. It is possible that the ^83^DI^85^SLWIEK^91^G motif did not involve the overall function of FimY to activate *fimZ*, and consequently *fimA*. Since there are three extra amino acid residues in the ^83^DI^85^SLWIEK^91^G motif of FimY than DxSxxG motif of other PilZ domains, we are not sure if these extra residues cause this motif a nonfunctional vestige. Therefore, it seems that the intact ^6^RRERHR^11^ motif may play a crucial role for the ultimate function of FimY.

There was a discrepancy between the number of fimbriae, yeast agglutination titer, and gene expression of *fimZ* and *fimA* in the R11A strain. This strain exhibited low *fimZ* and *fimA* expression in real‐time RT‐PCR, however, this did not correlate with the number of fimbriae and yeast agglutination titer, which were similar to the Δ*fimY* (*fimY*) strain. So far we have no reason to explain this phenomenon.

PHD revealed that N‐terminal of FimY possessed only an α‐helix, which is not likely to form a PilZ domain; however, the same analysis for MrkH, DgrA, and VCA0042 predicted six to seven β‐strands and a C‐terminal helix, consistent with a PilZ domain fold (Benach et al., 2007). Although FimY possesses ^6^RRxxx^11^R and ^83^Dx^85^Sxxxxx^91^G motifs, evidence to designate FimY as a PilZ domain protein was unavailable. FimY and PilZ domain proteins are not homologs. Nevertheless, the arginine at position 7 of the ^6^RRxxx^11^R motif has a crucial role in activating *fimZ* and consequently *fimA*, resulting in an expression of type 1 fimbriae in *S*. *typhimurium*.

## CONFLICT OF INTERESTS

The authors declare that they have no conflict of interest.

## AUTHOR CONTRIBUTIONS

Nan‐Ling Kuan drafted the manuscript and participated all the experiments. Kuang‐Sheng Yeh conceived this study and helped to draft the manuscript.

## ETHICS STATEMENT

Not required.

## Data Availability

All the data are provided in full in this manuscript.
